# One size does not fit all

**DOI:** 10.7554/eLife.02088

**Published:** 2014-01-21

**Authors:** Diethard Tautz

**Affiliations:** 1 **Diethard Tautz** is an *eLife* reviewing editor, and is at the Max Planck Institute for Evolutionary Biology, Plön, Germanytautz@evolbio.mpg.de

**Keywords:** ants, Formicidae, social insects, other

## Abstract

Comparing the anatomies of more than 100 different species of ants reveals that worker ants have enlarged necks, not seen in queens, that allow them to lift and carry objects many times heavier than themselves.

**Related research article** Keller RA, Peeters C, Beldade P. 2014. Evolution of thorax architecture in ant castes highlights trade-off between flight and ground behaviors. *eLife* **3**:e01539. doi: 10.7554/eLife.01539

**Image** The body shapes of queen ants (top) and worker ants (bottom) have evolved in different ways to reflect their different roles in the colony

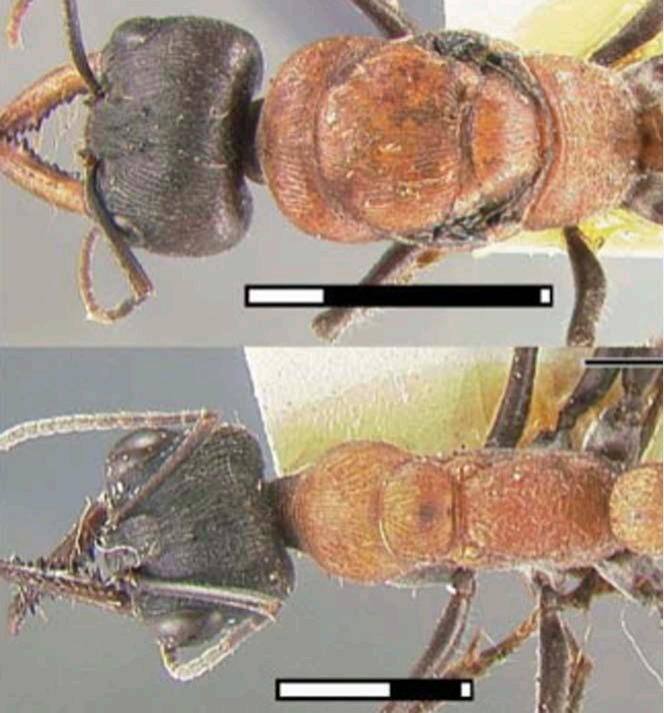

Ants are a spectacular evolutionary success (Hölldobler and Wilson, 1990). Different species have adapted their bodies and behaviour to exploit almost every conceivable ecological niche on Earth. In the Sahara, for example, silver ants search the scorching desert sands to scavenge corpses of heat-stricken animals (Wehner et al., 1992), while the leaf-cutting ants of the tropics harvest plant material to fertilize their fungus farms (Schultz and Brady, 2008). And further afield, the mangrove mud-nesting ant is believed unique amongst ant species in that it is able to swim underwater to forage in the tidal regions of northern Australia (Nielsen, 1997).

Discovering a new pattern within all this diversity would be a remarkable achievement—particularly considering that ants have been the subjects of scientific study for at least 140 years (Forel, 1874). Now, in *eLife*, Roberto Keller, Christian Peeters and Patrícia Beldade—who are based at the Instituto Gulbenkian de Ciência and the Université Pierre et Marie Curie—reveal that they have found one such pattern in the shape of the ant’s thorax (Keller et al., 2014).

Like all insects, an ant’s body is divided into three distinct parts: the head, the thorax, and the abdomen. Only queen ants and male ants have wings (which are attached to the thorax), whereas worker ants and other castes, such as the soldiers, do not have wings. Keller et al. have looked at 111 species of ants representing most ant subfamilies and discovered that workers are not simply scaled-down versions of queens that have lost their wings. Instead workers have a distinct thorax architecture with an enlarged muscle system to strengthen the neck and increase the range of motion of the head (Moll et al., 2010). This appears to be a key adaptation to allow ants to lift and carry objects or prey that are many times their own weight (Figure 1).

**Figure 1.**
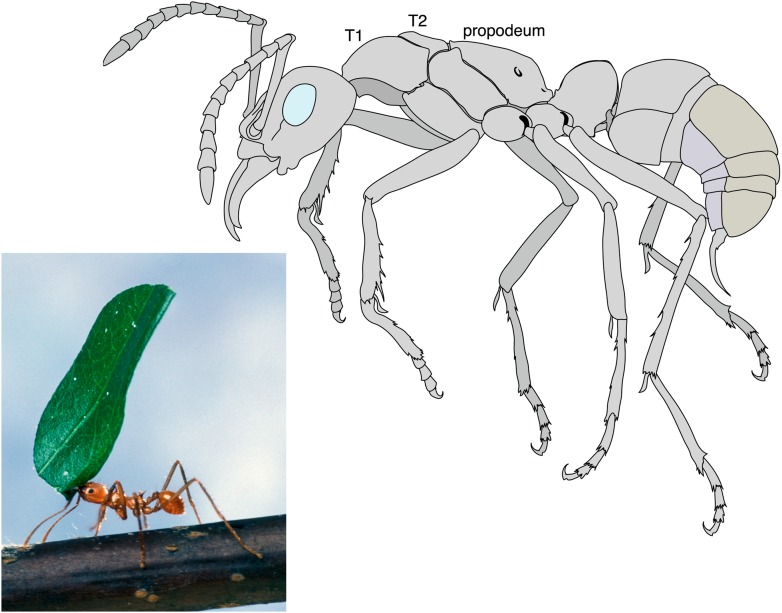
Leaf cutter ant showing the extraordinary strength of the worker´s neck. By measuring the lengths of the first and second segments of the thorax (T1 and T2), Keller et al. revealed that the workers of many ant species have an enlarged T1 or neck segment. The muscles within this segment are arranged in a way that has not been seen in other insects, and allow the worker ants to perform their amazing feats of strength. For example, some species are able to lift objects up to 90 times their own body weight. In ants the third segment of the thorax fuses with the first segment of the abdomen to form the propodeum.

Photograph: Scott Bauer, US Department of Agriculture. Illustration: Mariana Ruiz

In general, the anatomy of the insect thorax—three segments, called T1, T2 and T3—is thought to have contributed to their evolutionary success. Each segment has a pair of legs, and segments T2 and T3 can also support a pair of wings. Major modifications of the wings define whole insect families—such as the hardened forewings, or elytra, of the beetles (Coleoptera), and the severely reduced hind-wings, or halteres, of the flies (Diptera).

Ants and other social insects, such as bees and termites, belong in the order Hymenoptera. These insects usually have two pairs of wings: in ants, however, the wings on the T2 segment are much bigger than those on the T3 segment. Furthermore, in ants the T3 segment fuses with the first segment of the abdomen to form a structure called the propodeum (Figure 1). These features are well known and can be found in any textbook on insects. However, Keller et al. have uncovered a modification of the T1 segment that has been overlooked until now.

The approach used was rather straightforward: they simply measured the lengths of the three segments of the thorax, and then compared the results for queens and workers of the same species. They found that, across a large range of species, T2 is consistently reduced in workers, while T1 is enlarged. The reduction of T2 was not surprising, since this segment includes the bulk of the flight muscles, which are not needed in the wingless workers. However, the consistent enlargement of T1 merited further attention, and dissection of the thorax of workers from 19 ant species showed that this segment contains neck muscles and skeletal parts not seen in other insects.

On the other hand, the T1 anatomy of queens of the same species follows often the typical patterns known from other insects. Nevertheless, there are some interesting differences in thoracic anatomy of queens when assessed across the whole ant family tree. Queens of several ant species show an intermediate sized T1 segment, and these represent the ancestral lineages. Other species have a highly reduced T1 anatomy, which appears to have evolved independently in at least four major ant lineages, with a few transitions back to intermediate sized T1.

It is noteworthy that these anatomical differences correlate with the different strategies employed by queens when they are founding a new colony. A queen’s wing muscles allow her to fly away from the colony where she was born and to establish a new one at a distant location. Queens that establish new colonies on their own often have to forage outside the nest to gather food to raise their first brood, and these are indeed the species that tend to have an intermediate sized T1. This observation suggests a clear trade-off between investing in flying ability and investing in on-the-ground foraging ability. It is also a remarkable example for the power of evolution to find similar solutions to a given constraint, as different lineages have independently converged to the reduced T1 anatomy. At the same time, these findings imply that enlargement of the T1 segement is required for successful foraging, and since an enlarged T1 is found in workers of all the species examined, one can conclude that this is an ancestral feature of ant evolution. This means that it is probably one of the innovations that contributed to the evolutionary success of ants (Folgarait, 1998).

Keller et al. arrive at their conclusion with seemingly simple technical means, but they also draw on the large resources collected in museums and various specialized treatises on ant behaviour, as well as the meticulous reconstruction of the phylogeny of ants (Brady et al., 2006; Moreau et al., 2006). Nowadays, biological research is dominated by molecular approaches, knockouts and whole genome analysis—of course for good reason—but sometimes there is a danger that we might forget the richness and foundations of this discipline. The work of Keller, Peeters and Beldade serves to remind us that pure comparative anatomy and phylogenetic arguing remain as powerful as ever.
